# Allergy to seafood

**DOI:** 10.1186/2045-7022-1-S1-P28

**Published:** 2011-08-12

**Authors:** Natalia Ukleja-Sokolowska, Ewa Gawronska-Ukleja, Lukasz Sokolowski, Zbigniew Bartuzi

**Affiliations:** 1 Collegium Medicum in Bydgoszcz, Nicolaus Copernicus University in Torun, Allergology, Clinical Immunology and Internal Diseases, Bydgoszcz, Poland

## Background

Seafood is not a part of typical polish diet, although recently more and more households buy and eat shrimps, squids, octopus and moules. But are seafood allergens a problem for polish patients? Another interesting problem is cross-reactivity and co-reactivity between seafood and house dust mite allergens.

## The aim

Establish the frequency of allergy to seafood in patients treated because of allergy.

## Methods

The research was carried out in 86 patients treated in the Outpatients Clinic of Allergic Diseases. Every patient was interviewed for allergy history and family history of diseases. Every patient had:

1. Prick by prick (PbP) for seafood allergens (squid Illex argentinus and “Black tiger” shrimps Penaeus monodon, octopus Octopus vulgaris, moules Mytilus edulis ).

2. Skin prick test (SPT) to Dermatophagoides pteronissinus and Dermatophagoides farinae.

## Results

In the group of 86 patients 19 (22,09%) had positive PbP test to squids, 21 (24,42%) to shrimps, 12 (13,95%) to moules and 12 (13,95%) to octopus. 70 patients had positive SPT to D. pteronissinus or D. farinae. In this group 16 (22,86%) had positive PbP test to squids, 16 (22,86%) to shrimps, 10 (14,29%) to moules and 11 (15,71%) to octopus. In the group of patients without allergy to house dust mite (16 patients) 3 (18,75%) had positive PbP test to squids, 5 (31,25%) to shrimps, 2 (12,5%) to moules and 1 (6,25%) to octopus. Correlation between allergy to house dust mite and shrimps is 0,016, between house dust mite and squids is 0,081, between house dust mite and moules is 0,12, between house dust mite and octopus is 0,17.

## Conclusion

1. The most frequent positive prick by prick test was to shrimps (21 cases, 22,09%)

2. There is a weak correlation between allergy to house dust mite and octopus (0,17) and between house dust mite and moules (0,12), but no such correlation between house dust mite and shrimps or squids.

